# The potential consequences of grain-trade disruption on food security in the Middle East and North Africa region

**DOI:** 10.3389/fnut.2023.1239548

**Published:** 2023-10-16

**Authors:** Jaber Rahimi, Andrew Smerald, Hassane Moutahir, Mostafa Khorsandi, Klaus Butterbach-Bahl

**Affiliations:** ^1^Karlsruhe Institute of Technology (KIT), Institute of Meteorology and Climate Research, Atmospheric Environmental Research (IMK-IFU), Garmisch-Partenkirchen, Germany; ^2^Institut National de la Recherche Scientifique, Centre Eau Terre Environnement (INRS-ETE), Quebec City, QC, Canada; ^3^Pioneer Center Land-CRAFT, Department of Agroecology, Aarhus University, Aarhus, Denmark

**Keywords:** food, feed, protein, energy, trade, MENA

## Abstract

The Middle East and North Africa (MENA) region has seen remarkable population growth over the last century, outpacing other global regions and resulting in an over-reliance on food imports. In consequence, it has become heavily dependent on grain imports, making it vulnerable to trade disruptions (e.g., due to the Russia-Ukraine War). Here, we quantify the importance of imported grains for dietary protein and energy, and determine the level of import reductions at which countries are threatened with severe hunger. Utilizing statistics provided by the Food and Agriculture Organization (FAO), we employed a stepwise calculation process to quantify the allocation of both locally produced and imported grains between the food and feed sectors. These calculations also enabled us to establish a connection between feed demand and production levels. Our analysis reveals that, across the MENA region, 40% of total dietary energy (1,261 kcal/capita/day) and 63% of protein (55 g/capita/day) is derived from imported grains, and could thus be jeopardized by trade disruptions. This includes 164 kcal/capita/day of energy and 11 g/capita/day of protein imported from Russia and Ukraine. If imports from these countries ceased completely, the region would thus face a severe challenge to adequately feed its population. This study emphasizes the need for proactive measures to mitigate risks and ensure a stable food and feed supply in the MENA region.

## 1. Introduction

The Middle East and North Africa (MENA) region faces significant food security challenges due to limited arable lands and water resources (1), changing dietary habits (2, 3), and ongoing geopolitical conflict (4). All these challenges have been intensified by rapid population growth (5), which has exceeded 2% annually, and is higher than the global average for middle-income countries (1.3%) (2).

The MENA region struggles with a shortage of arable land, having only about 1.07 hectares of agricultural land per person (including 0.16 hectares of cropland per person), which is lower than the global average (0.20) (1). Additionally, the region faces severe water scarcity, with each person having access to only 9 percent of the world average, and in about two-thirds of the countries, more groundwater is abstracted than is naturally replaced (1). Despite water scarcity, the region has low water prices, due to allocating a significant portion of its resources to water subsidies (amounting to around 2% of GDP), and has an overall water productivity that is only half of the global average (6).

Food security and water scarcity are interconnected challenges in the MENA region (7). Geopolitical issues and insufficient arable land and water resources hinder domestic food production, making the region one of the least self-sufficient in terms of food (8, 9). Climate change makes these issues worse by causing less water to be available overall, increasing temperatures and speeding-up the rate at which water evaporates from the land (10–12).

As a result, MENA is highly dependent on imports (13, 14), especially of grains, where imports account for most of the supply (15). The region is thus highly vulnerable to trade disruptions that limit the global supply of grain and/or increase the price. These disruptions can have multiple underlying causes, including armed conflicts (either locally, as in Syria, or in important exporters, such as Ukraine), pandemics, surges in energy/fertilizer prices, coordinated crop failures across global breadbaskets (e.g., due to extreme events related to large scale droughts or floods) and trade wars.

Recent studies have already highlighted the adverse effects of grain import dependency in MENA (16, 17) and shown that it is expected to increase in the years ahead (18), potentially hindering the region’s ability to address its food insecurity. They have also highlighted the region’s vulnerability to trade disruptions caused by the war in Ukraine and COVID-19 (19, 20). For example, focusing on stable crops, Al-Saidi (21) has illustrated the varying levels of vulnerability to challenges stemming from these crises, in particular in Lebanon, Libya, Sudan, and Yemen. Furthermore, Ben Hassen and El Bilali (22) emphasized the potentially far-reaching and lasting consequences of conflict-related disruptions to global food and fertilizer markets, and the challenges this poses to the MENA region.

However, to our knowledge, there is a lack of research on the quantitative link between imported grains and dietary energy and protein supply, and therefore on the potential for trade disruption to affect diets. Quantifying this link is complicated by the dual use of imported grains as both human food and as livestock feed for the production of meat, milk and eggs. We address this by determining country-specific values for *per capita* energy and protein consumption that is attributable to imported grains, and assessing the potential impact on diets of trade disruptions. We are thus able to estimate the level of import reductions that would tip whole countries into a state of a severe lack of food.

## 2. Materials and methods

### 2.1. Study area

**Figure 1:**
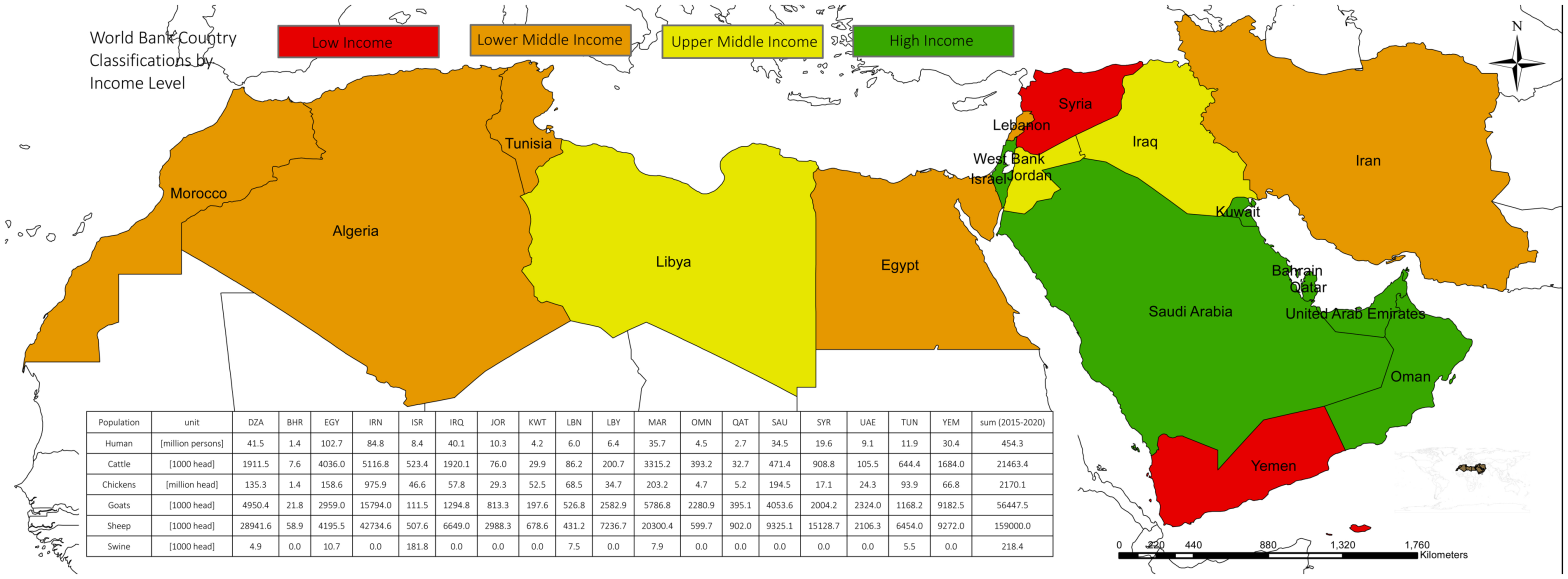
Income level classifications of MENA countries (2022–2023) according to the world bank (23). Human population and livestock numbers in MENA countries [average over 2015–2020; (15)].

The study area includes the 18 countries that make up the MENA region, which range from low-income countries such as Yemen to high-income oil-exporting countries such as the Arab states of the Persian Gulf. The countries are shown in Figure 1 along with their income class according to the World Bank classification (23). Over the past six decades (2020 compared to 1961), the livestock population (including cattle, dromedaries, sheep and goat) has more than doubled. During this period, meat production (from all livestock) also witnessed a surge from 1.1 million tons to 11.4 million tons. In the majority of MENA nations, the livestock sector’s share of the agricultural Gross Domestic Product (GDP) exceeds 30%, and, in certain countries, it reaches as high as 50%. The average livestock population during the period from 2015 to 2020 is presented in Figure 1.

### 2.2. Data analysis

The steps taken to address the specified research question are outlined below.

#### 2.2.1. Step 1: examining the sources of grain supply for food and feed sectors - local production and imported grains

**Figure 2:**
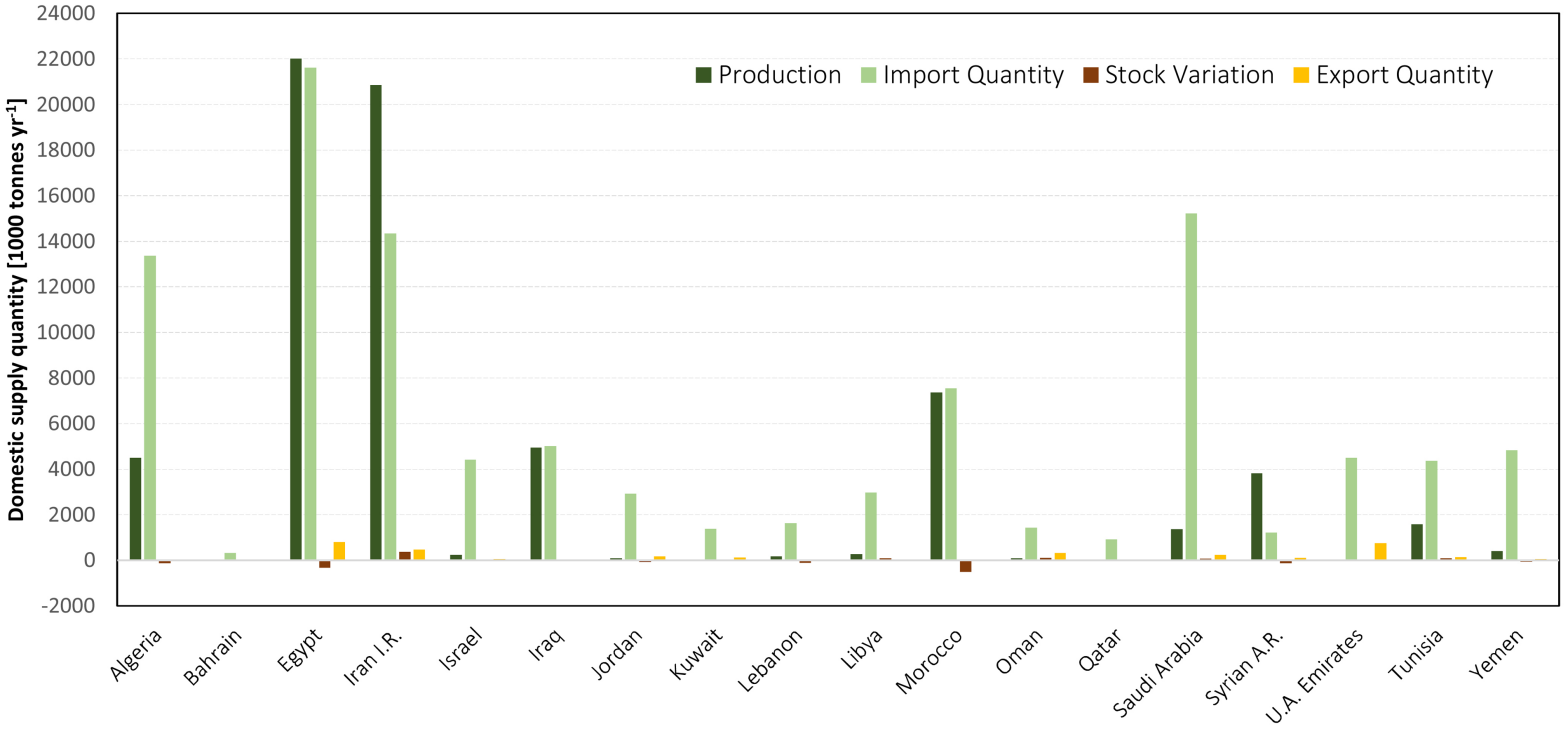
Domestic grain supply in MENA countries: insights from 2015 to 2020 FAOSTAT data (15).

Using the Food and Agriculture Organization’s (FAO) Food Balance Sheets (FBS) data on total production, export, import, and stock variations, the total domestic supply for each desired grain product was quantified, and the outcomes evaluated according to their allocation information [i.e., Food, Feed, and Other (non-food/industrial, seed and losses); Figure 2; Supplementary Figure 1]. In the case of grain import reductions, our strategy was to prioritize the allocation of imported grain to the food sector. We assumed no changes in both the quantity and the allocation of locally-produced grain. The FAO category ‘Other uses’ was deducted from locally-produced and imported grain according to its percentage of the total supply.

Data from FAO-FBS on the allocation of the 12 major grain crops in the region (Wheat and products, Rice and products, Barley and products, Maize and products, Rye and products, Oats, Millet and products, Sorghum and products, Soybeans, Rape and Mustard seed, Beans, and Peas) was used to see how grain production (averaged over 2015–2020) in a given country was split between food and feed usage. These grain products cover ∼93% of all crop-based feed usage in the region (and, at the same time ∼40% of crop-based food usage). We have then broken down the production to different usages (i.e., food vs. feed) using a crop allocation dataset (24, 25).

#### 2.2.2. Step 2: estimating feed demand using livestock production as a basis

In order to calculate livestock feed usage, country-specific data for meat, milk, egg, pork, and mutton production from cattle, sheep, goats, pigs/swine, and chicken for the period 2015–2020 was obtained from the FAOSTAT database (Supplementary Table 1). The production was then apportioned to grid cells based on the FAO’s spatially disaggregated livestock-source food supply dataset (26), which gives estimates of the share of production within a country. The dominant livestock production system (LPS) in each grid was then used to determine the total production of each livestock commodity in each LPS. Livestock production in each LPS was transformed into grain fodder and foliage demand via a fraction of grain forage in total feed dry matter and the feed conversion ratio (FCR) (27, 28). FCRs give the quantity of feed (kg-dry-matter) required to produce a given amount of livestock commodity at the herd level (i.e., they consider the need to also feed unproductive livestock, such as juveniles; Supplementary Table 2). This step is important, because in the subsequent stage we will need to backtrack and convert the feed deficit into livestock production.

#### 2.2.3. Step 3: conversion to energy and protein supplies and conducting scenario analysis

Information on the average dietary and nutritional value of each commodity (i.e., kcal/kg food and g protein per kg food; Tables 1, 2) was calculated to answer the question of what is the role of each food commodity in supplying energy and protein, and also to see how disturbing the balance of the grain trade can affect food and livestock production and consequently protein and energy supplied. For the scenario analysis, we have used the FAOSTAT trade matrix, the observatory of economic complexity (29), and the united nations commodity trade statistics (UN COMTRADE) (30) databases to obtain grain trade volumes to the region from Russia and Ukraine (Supplementary Figure 2).

**Table 1:** Food energy and protein supply from grain-dependent commodities (2015–2020).

Countries	Food energy supply (kcal/capita/day)	Food energy supply from grain-dependent commodities (share in the total)	Food energy supply from grain-crop-dependent commodities (share in the total)	Food energy supply from grain-livestock-dependent commodities (share in the total)	Food protein supply (g/capita/day)	Food protein supply from grain-dependent commodities (share in the total)	Food protein supply from grain-crop-dependent commodities (share in the total)	Food protein supply from grain-livestock-dependent commodities (share in the total)
DZA (Algeria)	3,430	0.64	0.52	0.12	90	0.83	0.56	0.27
BHR (Bahrain)	3,460	0.55	0.36	0.19	94	0.83	0.33	0.50
EGY (Egypt)	3,336	0.75	0.68	0.06	97	0.85	0.68	0.18
IRN (Iran)	3,059	0.59	0.51	0.08	84	0.77	0.52	0.24
ISR (Israel)	3,561	0.61	0.37	0.24	126	1.16	0.40	0.76
IRQ (Iraq)	2,602	0.55	0.50	0.05	64	0.63	0.49	0.14
JOR (Jordan)	2,663	0.46	0.37	0.09	68	0.62	0.36	0.26
KWT (Kuwait)	3,455	0.64	0.45	0.18	102	0.91	0.41	0.50
LBN (Lebanon)	2,884	0.52	0.42	0.10	70	0.72	0.43	0.29
LBY (Libya)	3,132	0.60	0.48	0.12	83	0.80	0.48	0.32
MAR (Morocco)	3,385	0.71	0.64	0.08	100	0.90	0.68	0.23
OMN (Oman)	2,947	0.53	0.38	0.16	86	0.70	0.33	0.38
QAT (Qatar)	3,459	0.62	0.44	0.17	101	0.88	0.39	0.48
SAU (Saudi Arabia)	3,307	0.63	0.51	0.12	90	0.84	0.48	0.36
SYR (Syrian Arab Republic)	2,822	0.51	0.41	0.10	74	0.64	0.42	0.22
UAE (United Arab Emirates)	3,068	0.56	0.42	0.14	83	0.77	0.42	0.35
TUN (Tunisia)	3,490	0.65	0.54	0.11	100	0.88	0.60	0.28
YEM (Yemen)	2,025	0.46	0.42	0.04	53	0.53	0.43	0.10
Average	3,116	0.59	0.47	0.12	86.84	0.79	0.47	0.33

**Table 2:** Food energy and protein supply from different commodities.

	Commodities	Countries																	
		DZA	BHR	EGY	IRN	ISR	IRQ	JOR	KWT	LBN	LBY	MAR	OMN	QAT	SAU	SYR	UAE	TUN	YEM
Energy (kcal/kg)	Grain crops	3,079	3,167	3,628	3,244	2,959	3,115	2,986	3,969	3,061	3,357	3,002	3,402	3,418	3,349	3,231	2,866	3,416	2,726
	Beef	1,817	1,749	1,528	1,992	1,982	1,482	1,843	2,179	2,061	1,783	1,887	1,896	1,901	2,142	2,695	1,677	1,890	2,666
	Milk	1,206	1,661	815	1,107	599	933	931	2,229	655	1,312	1,115	991	1,777	1,285	768	2,227	782	1,200
	Poultry	1,261	1,269	1,382	1,274	1,264	1,278	1,166	1,482	1,161	1,271	1,224	1,237	1,315	1,272	1,280	1,120	1,233	1,271
	Egg	1,231	1,441	1,421	1,421	1,412	1,423	1,353	1,671	1,284	1,428	1,231	1,414	1,499	1,448	1,420	1,277	1,246	1,422
	Pork	2,701	3,404	2,190	3,681	3,681	3,681	2,623	4,791	3,100	4,106	3,285	4,076	4,232	3,681	3,681	3,982	4,015	3,681
	Mutton and Goat meat	2,039	2,601	2,268	2,440	2,068	2,443	2,273	3,116	2,090	2,346	1,987	2,130	2,725	2,380	2,549	1,748	1,987	2,041
Protein (g per kg food)	Grain crops	128	126	141	127	118	118	134	127	127	139	129	136	124	125	135	119	145	101
	Beef	154	160	146	157	161	135	165	169	156	170	146	159	176	160	143	171	147	140
	Milk	68	103	50	76	44	54	61	121	43	68	78	54	97	87	41	114	46	92
	Poultry	112	129	128	130	151	133	131	126	131	130	126	126	127	131	133	124	135	130
	Egg	104	110	107	107	114	107	111	107	107	107	104	108	109	109	107	108	105	107
	Pork	146	117	124	124	97	124	124	111	131	152	183	116	115	124	124	137	124	124
	Mutton and Goat meat	133	135	144	139	133	139	138	135	143	141	136	137	135	141	137	137	136	148

## 3. Results

### 3.1. Current import dependency

**Figure 3:**
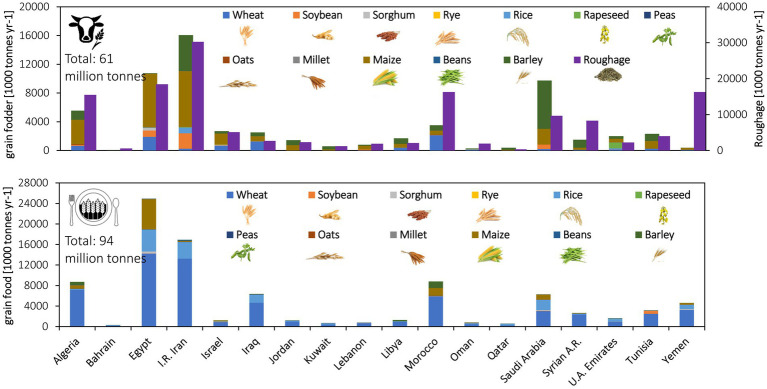
Grain utilization for food and fodder in MENA countries: 2015–2020 analysis.

**Figure 4:**
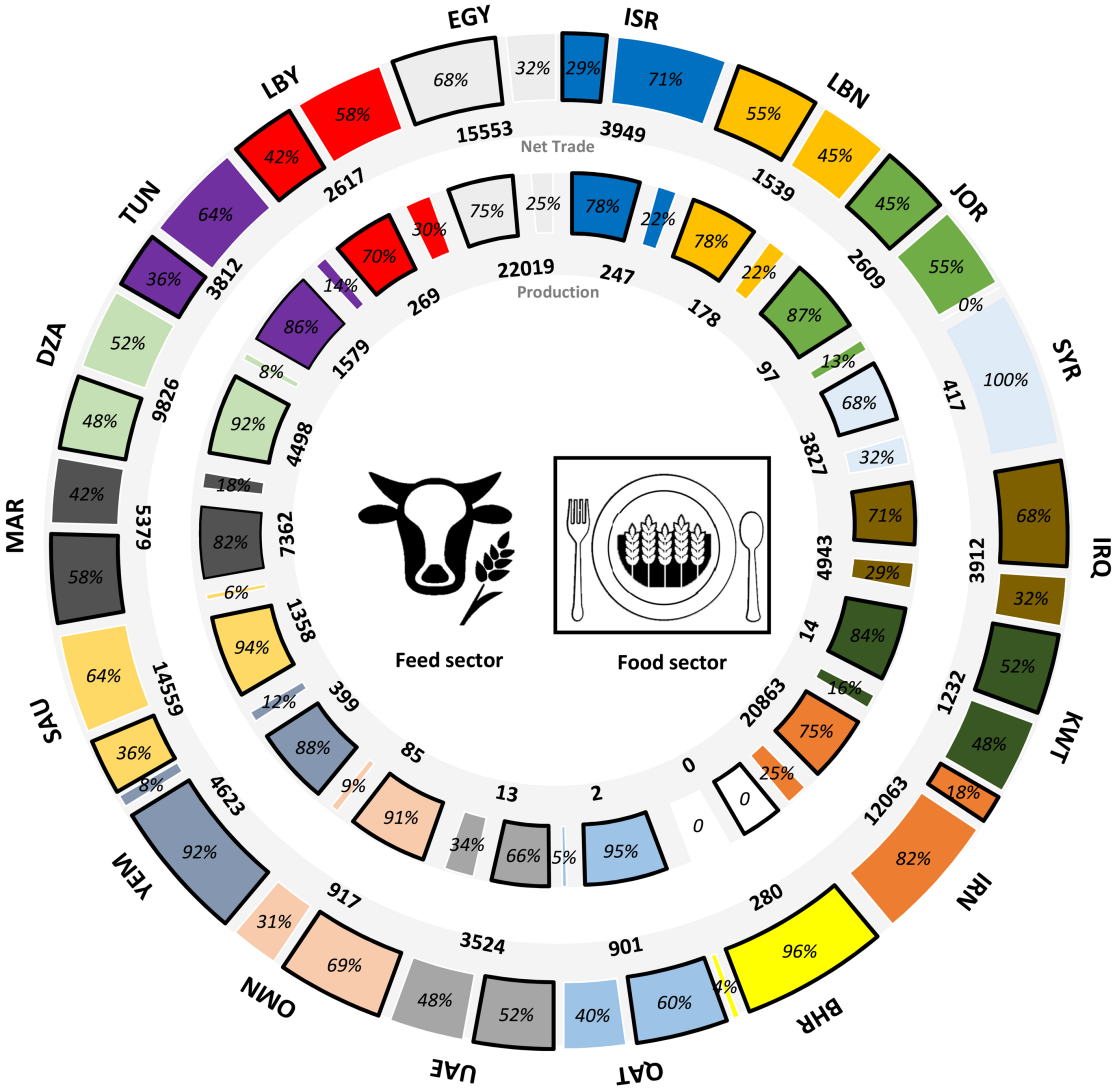
The allocation of the net grain trade and local production per country to the feed and food sectors in the MENA region; averaged over the 2015–2020 period (All numbers are in 1,000 tons).

The MENA region currently consumes 156 Million ton (Mt) of grain for food and feed per year (Figure 3), of which 68 Mt is produced locally, and 88 Mt. is imported. 94 Mt is directly consumed by humans, predominantly wheat (62 Mt), and the remaining 61 Mt. is fed to livestock, predominantly maize (28 Mt) and barley (20 Mt; Figure 3). Grains are particularly important for livestock production due to the importance of poultry in the region (poultry meat and eggs make up 29% of livestock production and consume 74% of total grain usage in the livestock sector) and constitute 33% of daily protein intake by weight and 12% of energy intake (compared to a global average of 31 and 15%; excluding offals, butter, and fat). Imports comprise 45% of food and 74% of feed (Figure 4).

The import dependency of individual countries is heterogeneous and can be split into three broad categories: (1) Syria (10% imported) and Iran (37%) rely primarily on domestic production; (2) Egypt (41%), Iraq (42%), and Morocco (44%) import approximately half their grains; and (3) The remaining 11 countries import the majority of the grain they consume. This includes relatively wealthy countries such as Bahrain, Kuwait, UAE, and Qatar, which are almost entirely dependent on imports, but also poor countries such as Yemen (Figure 1).

The vulnerability to trade disruption depends not just on the import ratio, but also on the grain dependency of diets and to what extent current diets exceed minimum requirements for energy [1,800 kcal/capita/day (31)] and protein [60 g/capita/day (32)] consumption. Across the MENA region, 59% of total dietary energy (1,832 out of 3,116 kcal/capita/day) and 79% of protein (69 out of 87 g/capita/day) is derived from grains, meaning 40% of energy and 63% of protein are from imported grains. Again, the region is heterogeneous. At current import levels, Yemen, on average, only just receives the daily minimum dietary requirements (2,025 kcal/capita/day and 53 g-protein/capita/day) and has a high grain dependency (92%), while Iran produces 63% of its grain demand.

### 3.2. Impact of grain-trade changes on food security

**Figure 5:**
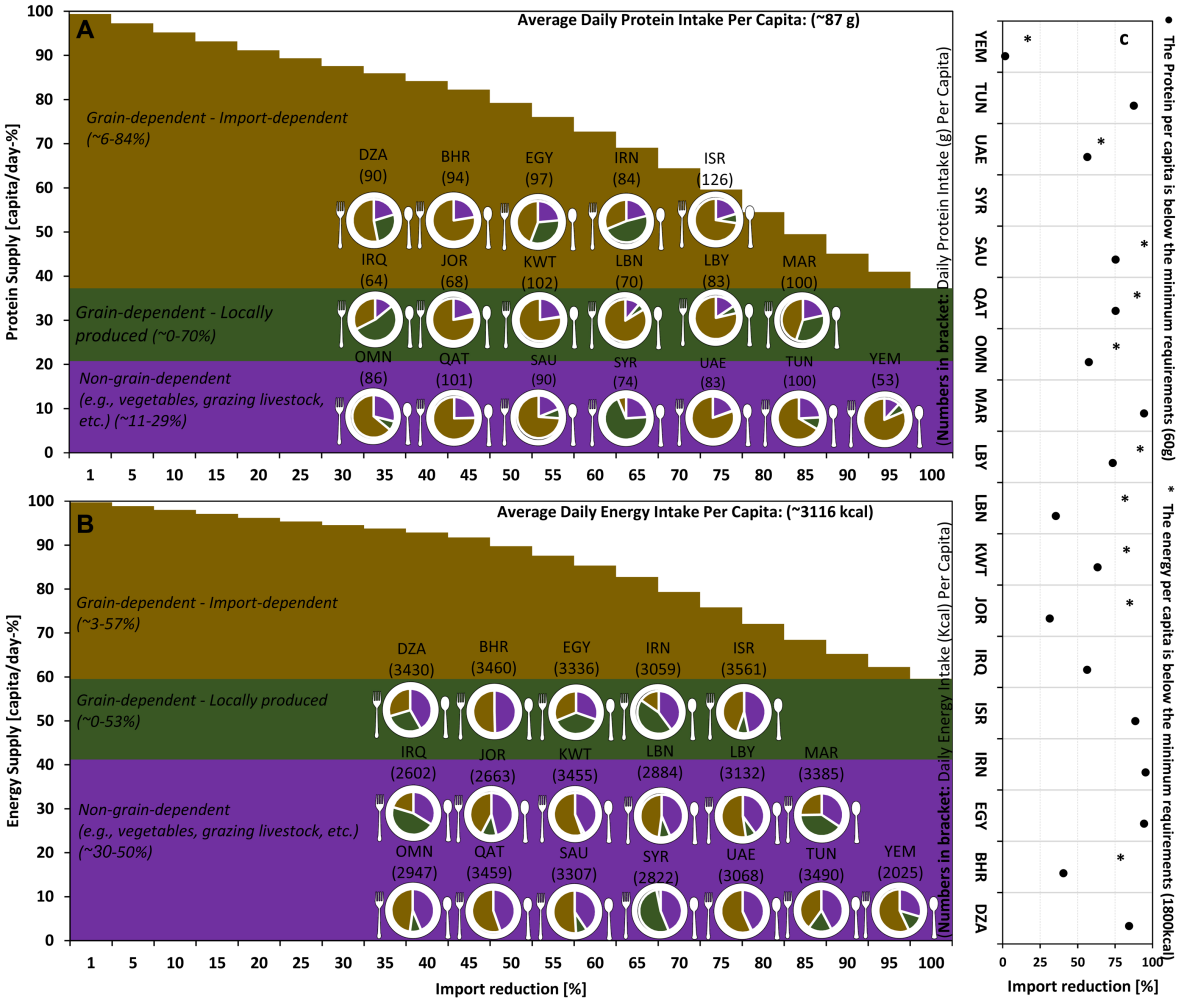
Possible consequences of change in total grain import to the MENA region on supplied protein and energy *per capita* per day as compared to the 2015–2020 average. c: % of import reduction at which dietary protein (∙) and energy (*) fall below the minimum *per capita* requirement.

Reducing grain imports to the MENA region will affect energy and protein supply differently, depending on how the impact is shared between the food and feed sectors. Our assumption is that food is prioritized, meaning that even small import reductions have a large effect on the livestock sector. Thus, dietary protein would fall faster than energy for small reductions in imports due to the importance of livestock for protein (Figure 5). With increasing import reductions, there comes a point at which the livestock sector may no longer receive imported grains, and further reductions fall exclusively on the food sector, resulting in a rapid decrease in dietary energy.

The level of import reduction at which the average energy or protein supply *per capita* falls below the minimum requirements varies between countries (Figure 5). For Yemen, dietary protein is already below the minimum, and dietary energy would fall below the minimum in the case of an 18% import reduction. On the other hand, Syria’s remarkable domestic grain production means that even if imports were reduced to zero, sufficient dietary protein and energy would remain available. Other countries fall between these two extremes, with, for example, Jordan falling below the minimum level for protein/energy at a 32/85% reduction and Oman at a 57/86% reduction. However, it should be noted that comparing the average dietary energy/protein to minimum requirements is a very conservative estimate for the point at which a country experiences severe food shortage. Due to the unequal distribution of food supplies, a large fraction of the population will experience shortages well before this point.

The actual impact of trade disruption on diets in the MENA region will also depend on the mitigation steps taken within each country. For short-term disruption, depleting grain reserves could allow diets to be maintained. However, the region as a whole has only 64 Mt. of reserves, equal to 73% of yearly grain imports. *Per capita* grain stock is estimated at 131 kg per person per year, ranging from 36 kg per person in Yemen to about 285 kg per person in Saudi Arabia (Supplementary Table 3). For longer-term reductions in import availability or affordability, adaptation would soften the impact on diets. However, lack of good quality arable and grazing land due to water and other constraints would limit the ability of most countries in the region to considerably increase domestic production. More drastic adaptions, such as large-scale emigration, would negatively affect people within the region and potentially on a broader scale.

### 3.3. Possible consequences of the Ukraine war

Russia and Ukraine are both large grain exporters and were in 2015–2020 responsible for 34% of imports to the MENA region (17% from each; Supplementary Figure 1). In the case of a complete loss of all imports from Russia and Ukraine, and assuming no substitution from elsewhere, dietary protein in MENA countries would be reduced by 11 g/capita/day and dietary energy by 164 kcal/capita/day. This would be sufficient to tip Lebanon and Yemen 51 and 47% below the minimum protein requirements, even in the case of only Ukrainian grain being unavailable. There would also be severe consequences for Israel, Libya, Oman, and Tunisia, which would approach the minimum dietary requirements, potentially leading to a large fraction of the population experiencing severe hunger (Table 3).

**Table 3:** Potential energy and protein loss (%) due to complete loss of all imports from Russia, Ukraine, and both.

Countries	Potential loss due to complete loss of all imports from Russia		Potential loss due to complete loss of all imports from Ukraine		Potential loss due to complete loss of all imports from Russia and Ukraine	
	Protein (%)	Energy (%)	Protein (%)	Energy (%)	Protein (%)	Energy (%)
DZA	0.31	0.13	1.99	0.81	2.30	0.94
BHR	0.13	0.05	1.17	0.51	1.30	0.57
EGY	5.38	2.03	5.38	2.03	10.75	4.06
IRN	3.73	1.31	2.90	1.02	6.63	2.32
ISR	6.92	2.88	21.65	8.39	28.57	11.28
IRQ	0.03	0.01	0.05	0.02	0.09	0.03
JOR	8.94	2.82	3.83	1.21	12.76	4.03
KWT	3.31	1.45	1.47	0.64	4.77	2.09
LBN	21.96	8.02	36.15	17.17	58.11	25.19
LBY	5.02	2.12	13.54	5.72	18.56	7.84
MAR	2.45	0.94	7.03	2.70	9.48	3.65
OMN	11.92	5.95	2.24	1.12	14.17	7.07
QAT	1.54	0.63	1.55	0.64	3.09	1.27
SAU	4.57	1.76	3.94	1.52	8.51	3.28
SYR	1.60	0.70	1.11	0.49	2.71	1.19
UAE	2.94	1.18	1.12	0.45	4.06	1.63
TUN	1.62	0.67	11.45	4.71	13.07	5.38
YEM	23.65	12.26	15.91	6.25	39.57	18.51

Finally, it should be remembered that grain import reductions may either be accompanied by a decrease in domestic supply (for example, due to an increase in energy/fertilizer prices), or may cause a drop in domestic supply by sparking armed conflict. In either case, dietary protein/energy availability would take an additional hit, further pushing countries toward the precipice of severe food shortages. For example, one of the short-term causes of the Arab Spring of 2010–2013 is widely believed to be a sharp rise in food prices (33), and the resulting civil wars in Syria and Libya significantly reduced domestic grain production (34). This study shows the vulnerability of the region to reductions in grain imports. Possible mitigation actions would be to build up stocks, reduce the dependency on animal-derived food and to diversify the source of imports.

## 4. Discussion

The MENA region heavily relies on grain as a significant source of calories and protein for its population. This dependence on grain imports poses a risk to domestic food security, as disruptions in grain trade can lead to reduced availability of calories and protein, potentially pushing some MENA countries below the minimum *per capita* requirements. Disruptions in exports from the Black Sea region, along with elevated prices, might accelerate the ongoing critical food situation in these regions.

Strategies for reducing the region’s vulnerability to trade disruptions are country specific, and depend on the country’s current reliance on imported grain, as well as logistical complexities. Possible strategies include trade control and diversification, subsidizing domestic agriculture, fostering international cooperation and aid initiatives, as well as establishing collaborative mechanisms within the region (21).

Our study is part of a growing body of literature that has begun to investigate the impact of trade disruptions on food security, often motivated by the Russian-Ukrainian war and the COVID-19 pandemic. Previous studies predominantly focused on price dynamics in specific regions [e.g. (35, 36)] or developed indicators for the vulnerability of regions to trade shocks [e.g. (37)]. For example, Prantner and Al-Naggar (38) investigated the consequences of the Ukraine-Russia conflict in Yemen and warned that, given the current circumstances, an extended food crisis and potential suspension of humanitarian aid could endanger the survival of millions. Abay et al. (13) classified countries into 10 risk categories, with a particular focus on the short-term impact of crises on regional and national food security. Our results are qualitatively similar to these findings.

However, our research diverges from earlier investigations in two critical aspects. Firstly, we provide a quantitative assessment of the repercussions of trade disruption on both the food and feed sectors, and the resulting reductions in dietary energy and protein consumption. Secondly, our study offers a regional overview of the entire MENA region, which allows countries individual circumstances to be put in a regional context, and highlights possibilities for collaboration.

## 5. Conclusion

The recent global food crisis, whose origins include higher costs of essential resources such as fuel and fertilizers, climate change, COVID-19 and conflicts, has hit many countries hard. This includes many countries in the MENA region, some of which were already facing critical food shortages.

Here we have quantified the extent to which MENA countries rely on imported grains, both in general and from Russia and Ukraine in particular. This included determining the importance of imports for energy and protein supply, and identifying at what level of import reduction countries may face severe food shortages. The country-specific analysis shows strong variation in the vulnerability of different MENA nations to import reductions and provides a starting point for understanding how adaption and mitigation measures could make the region more food secure.

It would be valuable in future work to analyze the food-feed system in the MENA region dynamically, considering how economic and societal drivers could spur adaption to reductions in the quantity of imported grain. For example, via increasing the quantity of imported animal products, conversion of suitable grazing land to arable production or reduction in food waste. Alternatively, the effect of trade disruptions on the food supply could be amplified if they lead to economic collapse, for example due to civil war. The ability of countries to adapt will critically depend on the strength and duration of trade disruptions, as well as economic and societal factors, land availability and government policies.

## Data availability statement

The original contributions presented in the study are included in the article/Supplementary material, further inquiries can be directed to the corresponding author.

## Author contributions

JR and AS conceived and designed the study. JR pre-processed the data and performed the analyses. JR wrote the first draft with support from AS while HM, MK, and KB-B contributed to improving the manuscript. All authors contributed to the article and approved the submitted version.
