# Supplementary material for: The potential consequences of grain-trade disruption on food security in the Middle East and North Africa region
Source: Front Nutr. 2023 Oct 16;10:1239548. doi: 10.3389/fnut.2023.1239548 (PMC10613703; doi:10.3389/fnut.2023.1239548)
Supplement: Supplementary file 1 [file Data_Sheet_1.docx]

Supplementary Material

The potential consequences of grain-trade disruption on food security in the Middle East and North Africa region

Jaber Rahimi^1*^, Andrew Smerald^1^, Hassane Moutahir^1^, Mostafa Khorsandi^2^, Klaus Butterbach-Bahl^1,3^

^1^ Karlsruhe Institute of Technology (KIT), Institute of Meteorology and Climate Research, Atmospheric Environmental Research (IMK-IFU), Garmisch-Partenkirchen, Germany

^2^ Institut National de la Recherche Scientifique, Centre Eau Terre Environnement (INRS-ETE), 490 De la Couronne St., Quebec City, Quebec, G1K 9A9, Canada

^3^ Pioneer Center Land-CRAFT, Department of Agroecology, Aarhus University, Aarhus, Denmark

*** Correspondence:**Corresponding Author
Jaber.rahimi@kit.edu

# Supplementary Figures and Tables

## Supplementary Figures


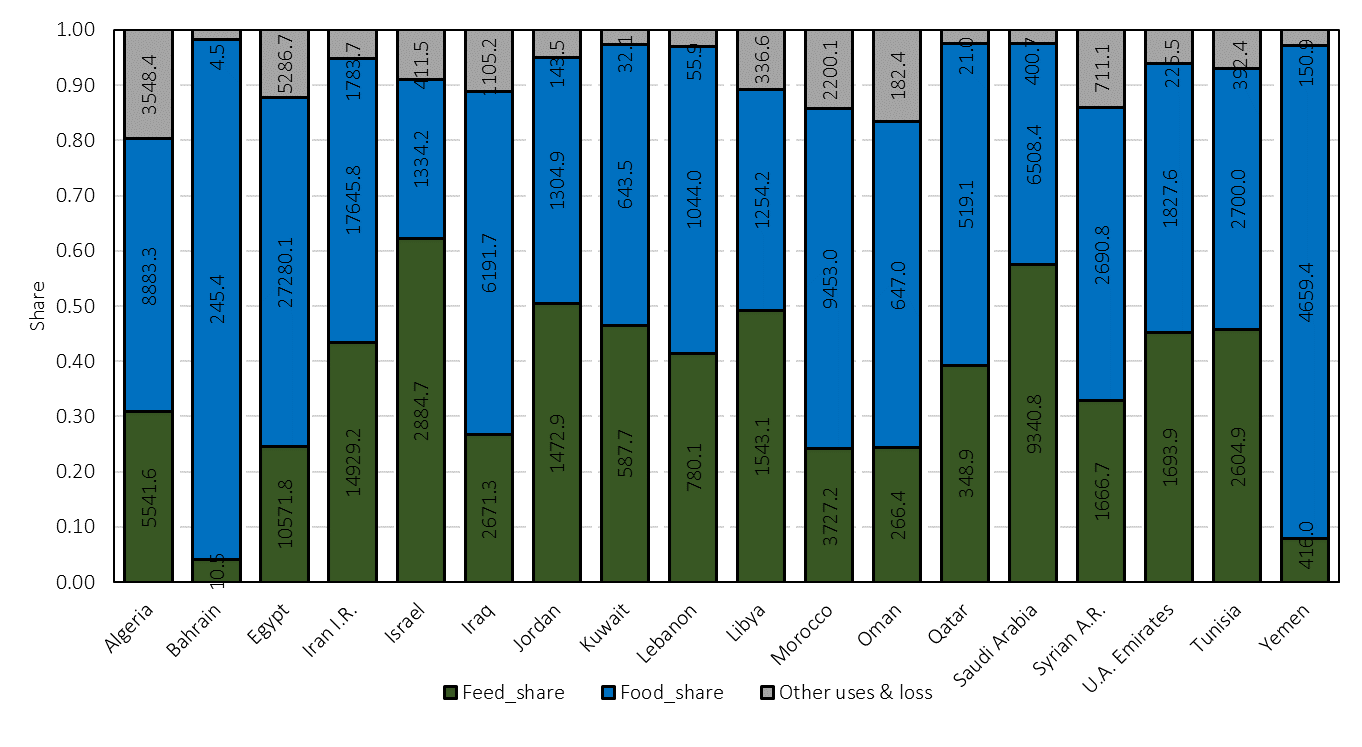


**Supplementary Figure 1:** Share of grains used for domestic use as food, livestock feed, and other uses & losses (numbers are in 1000 tonnes yr^-1^): insights from 2015-2020 FAOSTAT data (Source: FAOSTAT 2023).


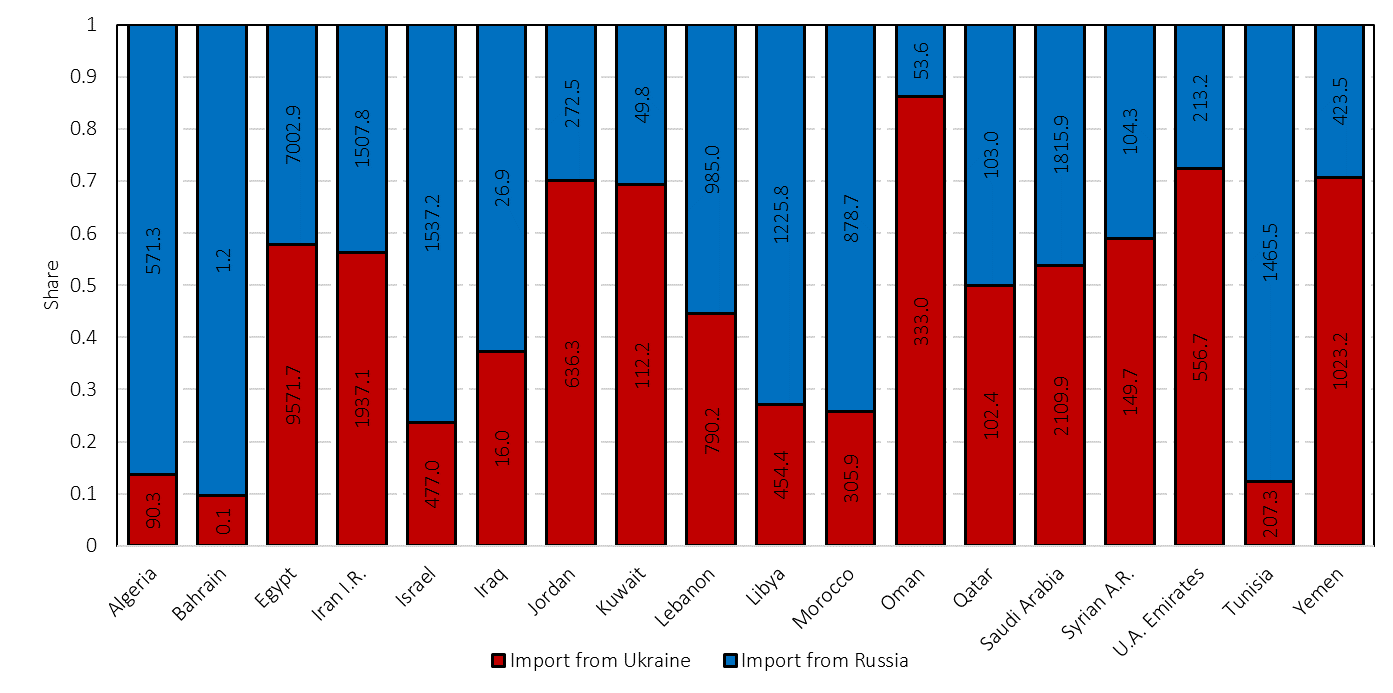


**Supplementary Figure 2:** Grain import from Ukraine and Russia to the MENA countries (numbers are in 1000 tonnes yr^-1^): insights from 2015-2020 FAOSTAT data (Source: OEC 2023)

## Supplementary Tables

**Supplementary Table 1:** Livestock production by commodities in MENA countries, average over 2015-2020 period (Source: FAOSTAT 2023).

| **production  [1000 tonnes yr^-1^]** | **Beef** | **Milk** | **Poultry** | **Egg** | **Pork** | **Mutton & Goat meat** |
| --- | --- | --- | --- | --- | --- | --- |
| DZA | 167.56 | 2064.92 | 285.57 | 221.87 | 0.13 | 311.83 |
| BHR | 1.26 | 9.27 | 9.19 | 2.45 | 0.00 | 23.46 |
| EGY | 377.60 | 2889.02 | 1294.03 | 321.80 | 0.87 | 72.29 |
| IRN | 262.62 | 4666.14 | 2188.49 | 460.65 | 0.00 | 204.96 |
| ISR | 119.65 | 845.95 | 557.29 | 83.29 | 11.92 | 28.42 |
| IRQ | 34.53 | 404.33 | 109.90 | 27.56 | 0.00 | 46.50 |
| JOR | 32.16 | 183.72 | 240.01 | 37.05 | 0.00 | 33.29 |
| KWT | 1.95 | 40.83 | 55.12 | 46.48 | 0.00 | 48.71 |
| LBN | 50.88 | 198.83 | 99.56 | 28.12 | 0.71 | 7.07 |
| LBY | 7.82 | 157.40 | 123.75 | 45.03 | 0.00 | 44.14 |
| MAR | 288.63 | 2559.78 | 644.74 | 227.39 | 0.62 | 206.80 |
| OMN | 21.34 | 147.32 | 8.09 | 14.26 | 0.00 | 36.63 |
| QAT | 1.78 | 7.73 | 15.45 | 4.47 | 0.00 | 11.57 |
| SAU | 47.96 | 1295.76 | 728.66 | 201.13 | 0.00 | 116.58 |
| SYR | 88.22 | 982.22 | 110.48 | 76.52 | 0.00 | 111.52 |
| UAE | 18.40 | 55.75 | 51.09 | 34.87 | 0.00 | 63.31 |
| TUN | 56.84 | 1024.78 | 134.04 | 70.59 | 0.13 | 61.99 |
| YEM | 177.45 | 403.77 | 200.60 | 43.70 | 0.00 | 154.62 |
| **sum** | 1756.64 | 17937.51 | 6856.07 | 1947.24 | 14.38 | 1583.70 |

**Supplementary Table 2:** Percentage of production, feed conversion efficiencies, and in each livestock production systems.

| **livestock category** | **percentage of production in each livestock production systems (Source: calculated based on** FAO 2011) | **feed conversion efficiencies – in kg of feed (dry mass) per kg of output** | **fraction of grain fodder in total feed dry matter** |
| --- | --- | --- | --- |
|  |  | **(Source: Mekonnen and Hoekstra 2012; Herrero et al. 2013)** | |
| beef_cattle_grazing | 50.33 | 97 | 0.03 |
| beef_cattle_mixed | 32.87 | 41 | 0.06 |
| beef_cattle_industrial | 16.80 | 13 | 0.41 |
| dairy_cattle_grazing | 60.78 | 3.3 | 0.24 |
| dairy_cattle_mixed | 28.23 | 1.9 | 0.39 |
| dairy_cattle_industrial | 10.99 | 1.5 | 0.65 |
| broiler_chickens_grazing | 58.50 | 8.9 | 0.93 |
| broiler_chickens_mixed | 28.69 | 4 | 0.99 |
| broiler_chickens_industrial | 12.81 | 2 | 1.00 |
| layer_chickens_grazing | 57.88 | 9.7 | 0.77 |
| layer_chickens_mixed | 29.17 | 4.6 | 0.97 |
| layer_chickens_industrial | 12.95 | 2.3 | 1.00 |
| pigs_grazing | 40.72 | 15.4 | 0.60 |
| pigs_mixed | 42.25 | 8.2 | 0.92 |
| pigs_industrial | 17.03 | 4.4 | 0.95 |
| sheep_goats_grazing | 62.55 | 33 | 0.01 |
| sheep_goats_mixed | 27.73 | 16.7 | 0.03 |
| sheep_goats_industrial | 9.72 | 7.3 | 0.22 |

**Supplementary Table 3:** Per capita grain stock in MENA countries, average over 2015-2020 period (Source: FAOSTAT 2023).

| **Countries** | **kg of grain stock per person per year** |
| --- | --- |
| DZA | 112 |
| BHR | 85 |
| EGY | 120 |
| IRN | 179 |
| ISR | 109 |
| IRQ | 126 |
| JOR | 56 |
| KWT | 134 |
| LBN | 87 |
| LBY | 190 |
| MAR | 169 |
| OMN | 65 |
| QAT | 169 |
| SAU | 285 |
| SYR | 64 |
| UAE | 107 |
| TUN | 273 |
| YEM | 36 |
